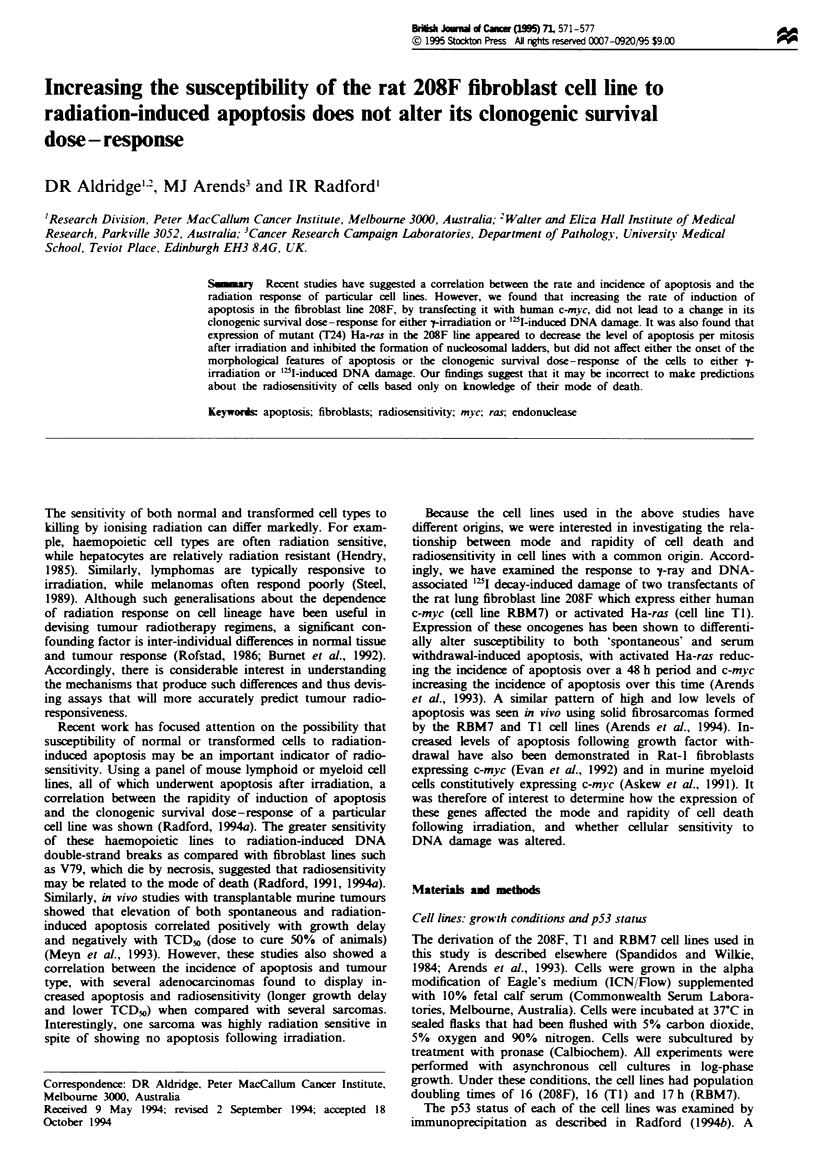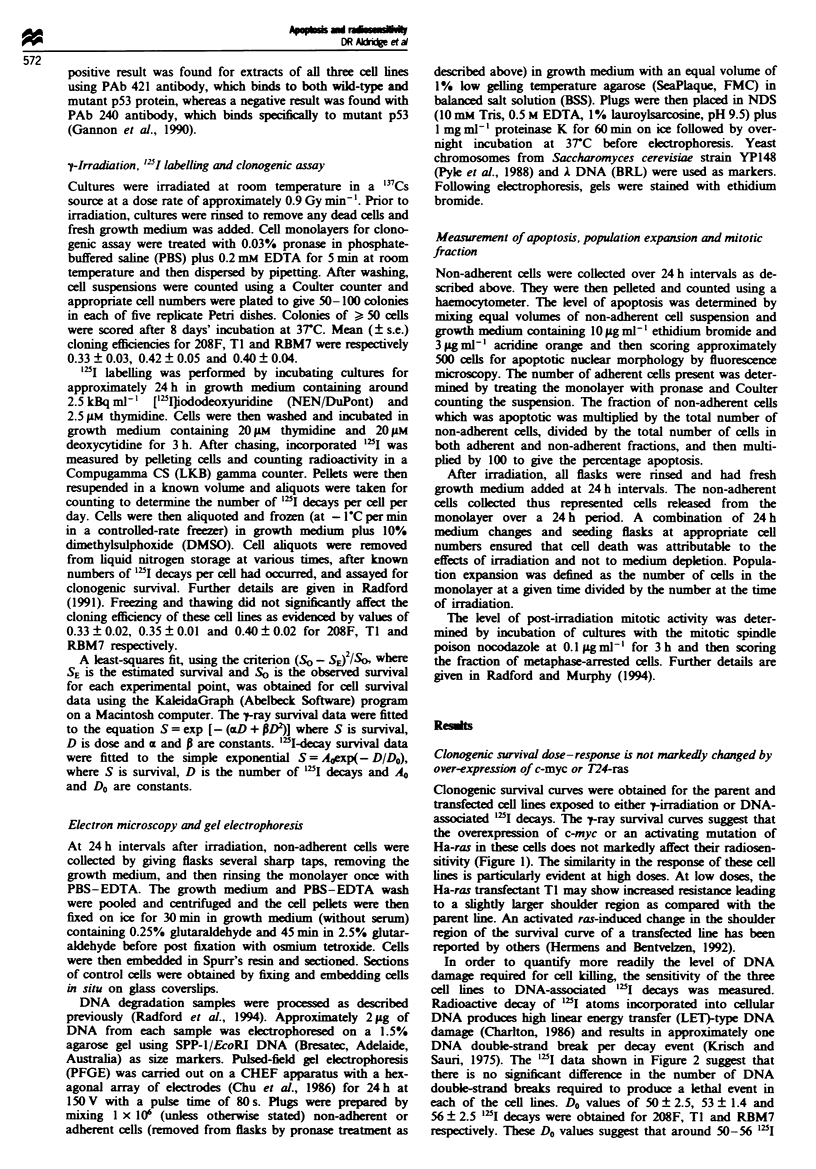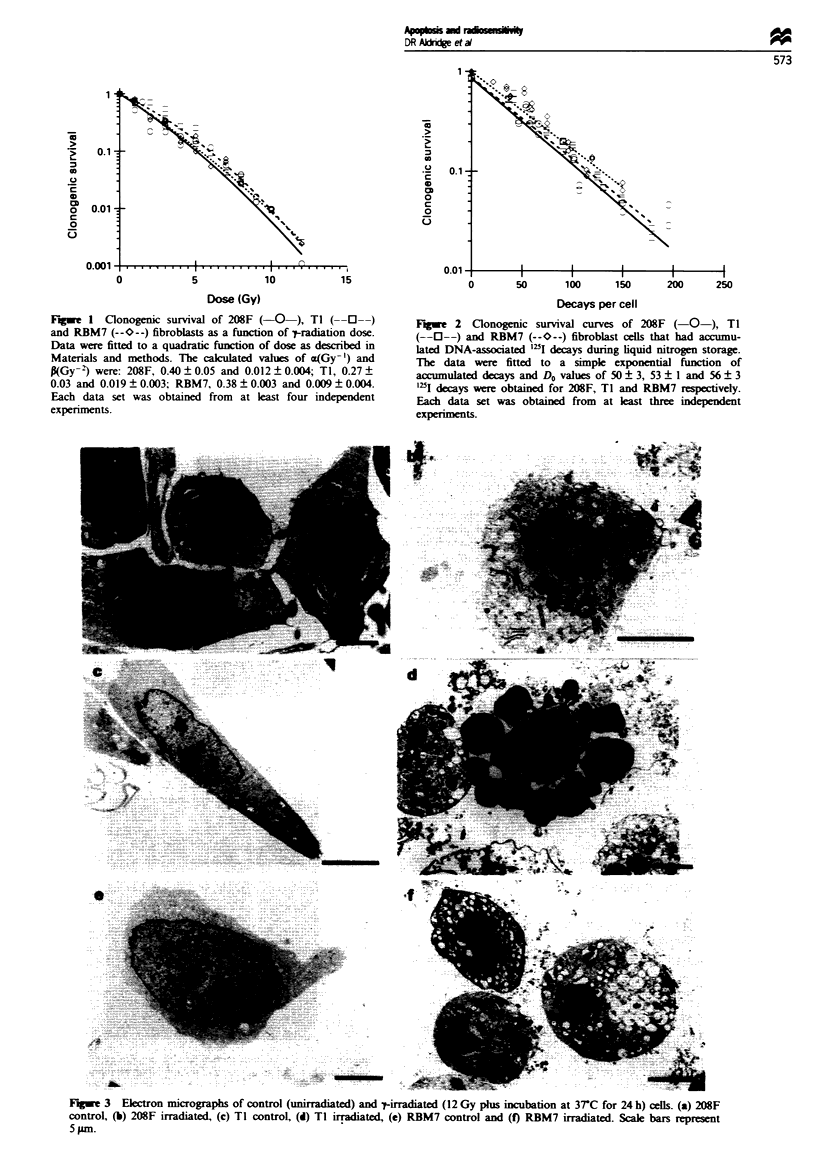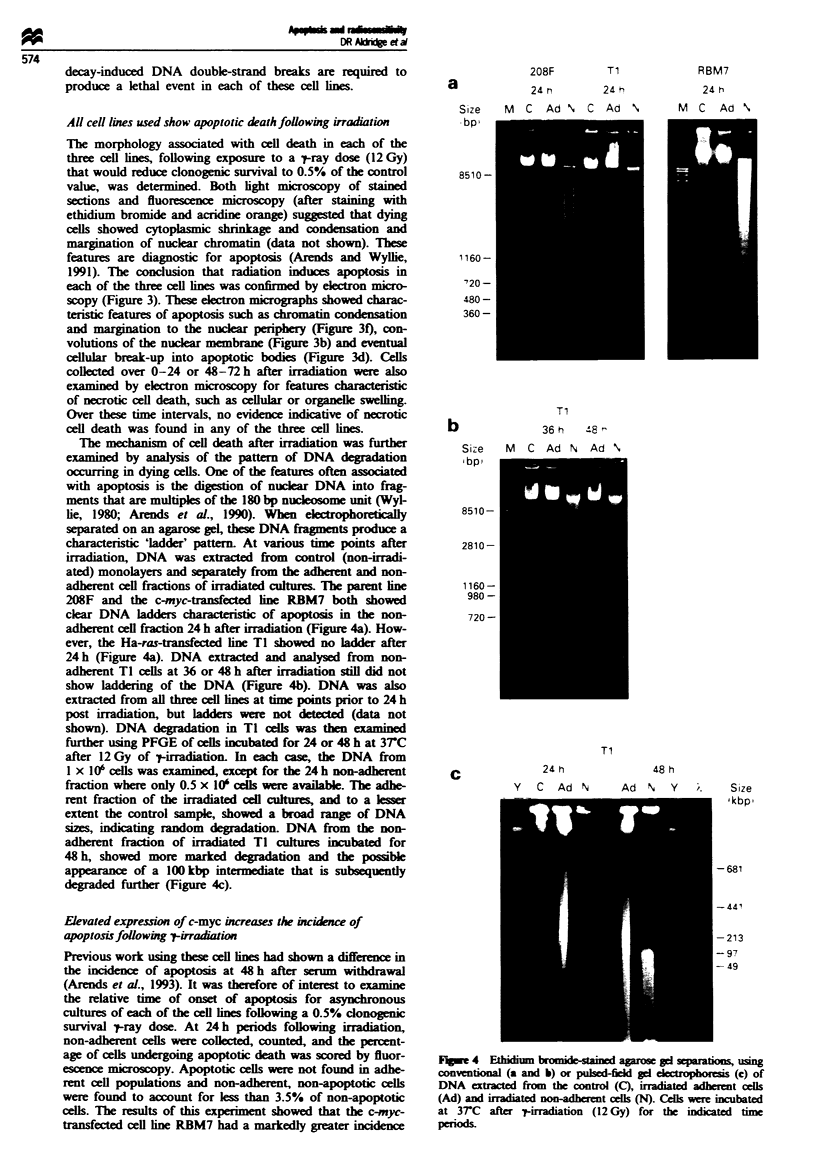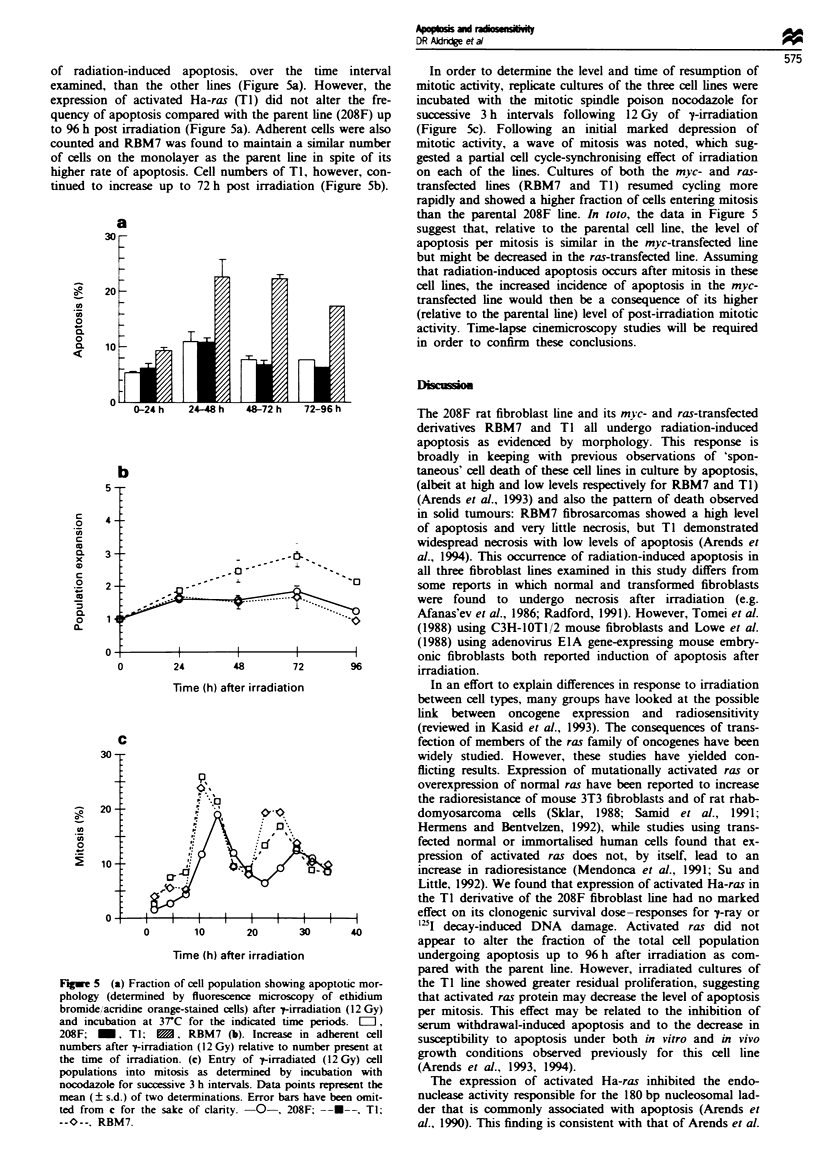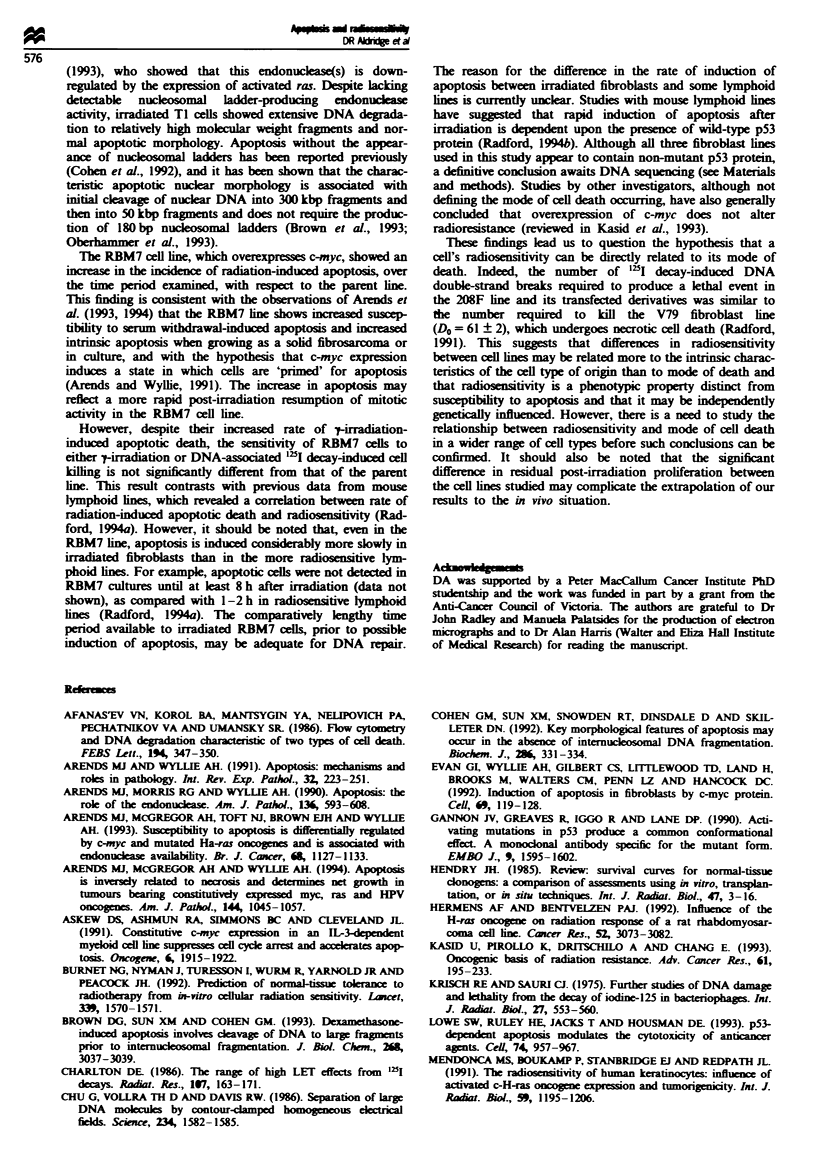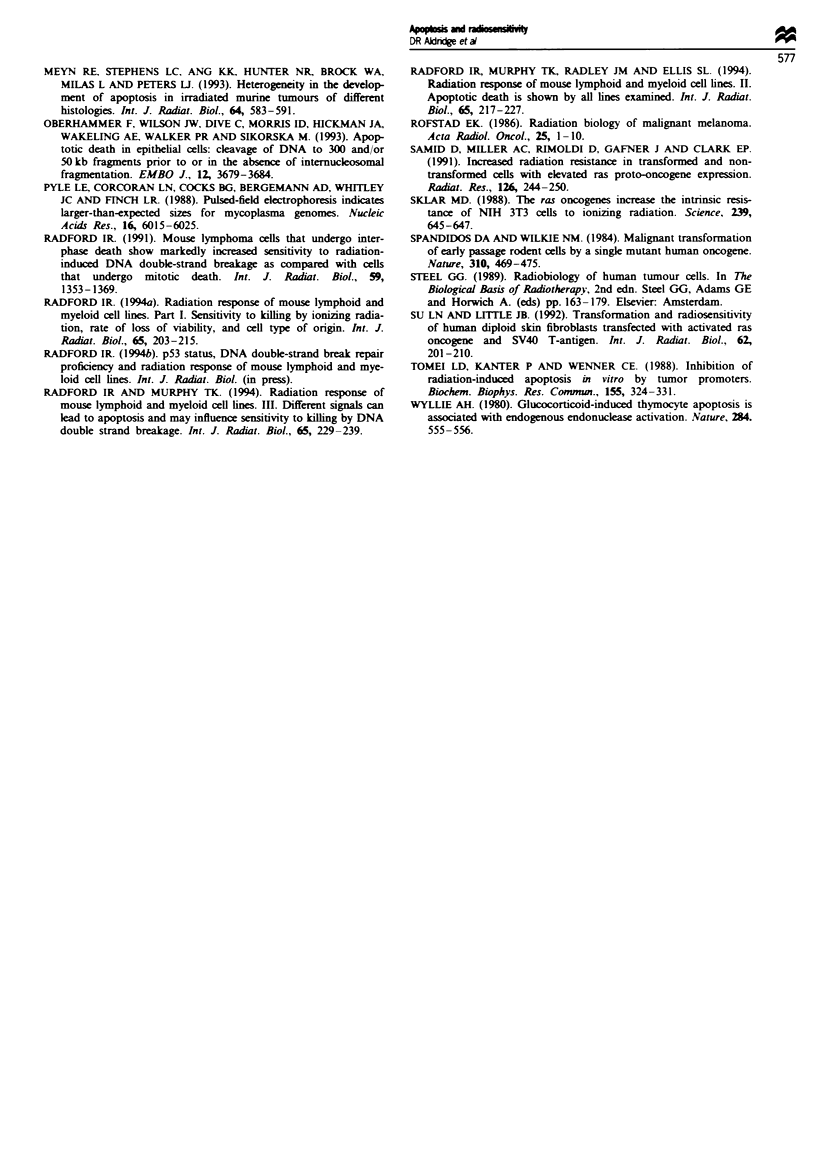# Increasing the susceptibility of the rat 208F fibroblast cell line to radiation-induced apoptosis does not alter its clonogenic survival dose-response.

**DOI:** 10.1038/bjc.1995.111

**Published:** 1995-03

**Authors:** D. R. Aldridge, M. J. Arends, I. R. Radford

**Affiliations:** Research Division, Peter MacCallum Cancer Institute, Melbourne, Australia.

## Abstract

**Images:**


© 1995 Stockton Press All rights reserved 0007-0920/95 $9.00

### Increasing the susceptibility of the rat 208F fibroblast cell line to radiation-induced apoptosis does not alter its clonogenic survival dose–response

DR Aldridge[1,2], MJ Arends[3] and IR Radford[1]

[1]*Research Division, Peter MacCallum Cancer Institute, Melbourne 3000, Australia;* [2]*Walter and Eliza Hall Institute of Medical Research, Parkville 3052, Australia;* [3]*Cancer Research Campaign Laboratories, Department of Pathology, University Medical School, Teviot Place, Edinburgh EH3 8AG, UK.*

**Summary** Recent studies have suggested a correlation between the rate and incidence of apoptosis and the radiation response of particular cell lines. However, we found that increasing the rate of induction of apoptosis in the fibroblast line 208F, by transfecting it with human c-*myc*, did not lead to a change in its clonogenic survival dose–response for either γ-irradiation or $^{125}$I-induced DNA damage. It was also found that expression of mutant (T24) Ha-*ras* in the 208F line appeared to decrease the level of apoptosis per mitosis after irradiation and inhibited the formation of nucleosomal ladders, but did not affect either the onset of the morphological features of apoptosis or the clonogenic survival dose–response of the cells to either γ-irradiation or $^{125}$I-induced DNA damage. Our findings suggest that it may be incorrect to make predictions about the radiosensitivity of cells based only on knowledge of their mode of death.

**Keywords:** apoptosis; fibroblasts; radiosensitivity; *myc*; *ras*; endonuclease

The sensitivity of both normal and transformed cell types to killing by ionising radiation can differ markedly. For example, haemopoietic cell types are often radiation sensitive, while hepatocytes are relatively radiation resistant (Hendry, 1985). Similarly, lymphomas are typically responsive to irradiation, while melanomas often respond poorly (Steel, 1989). Although such generalisations about the dependence of radiation response on cell lineage have been useful in devising tumour radiotherapy regimens, a significant confounding factor is inter-individual differences in normal tissue and tumour response (Rofstad, 1986; Burnet *et al.*, 1992). Accordingly, there is considerable interest in understanding the mechanisms that produce such differences and thus devising assays that will more accurately predict tumour radioresponsiveness.

Recent work has focused attention on the possibility that susceptibility of normal or transformed cells to radiation-induced apoptosis may be an important indicator of radiosensitivity. Using a panel of mouse lymphoid or myeloid cell lines, all of which underwent apoptosis after irradiation, a correlation between the rapidity of induction of apoptosis and the clonogenic survival dose–response of a particular cell line was shown (Radford, 1994*a*). The greater sensitivity of these haemopoietic lines to radiation-induced DNA double-strand breaks as compared with fibroblast lines such as V79, which die by necrosis, suggested that radiosensitivity may be related to the mode of death (Radford, 1991, 1994*a*). Similarly, *in vivo* studies with transplantable murine tumours showed that elevation of both spontaneous and radiation-induced apoptosis correlated positively with growth delay and negatively with $TCD_{50}$ (dose to cure 50% of animals) (Meyn *et al.*, 1993). However, these studies also showed a correlation between the incidence of apoptosis and tumour type, with several adenocarcinomas found to display increased apoptosis and radiosensitivity (longer growth delay and lower $TCD_{50}$) when compared with several sarcomas. Interestingly, one sarcoma was highly radiation sensitive in spite of showing no apoptosis following irradiation.

Because the cell lines used in the above studies have different origins, we were interested in investigating the relationship between mode and rapidity of cell death and radiosensitivity in cell lines with a common origin. Accordingly, we have examined the response to γ-ray and DNA-associated $^{125}$I decay-induced damage of two transfectants of the rat lung fibroblast line 208F which express either human c-*myc* (cell line RBM7) or activated Ha-*ras* (cell line T1). Expression of these oncogenes has been shown to differentially alter susceptibility to both 'spontaneous' and serum withdrawal-induced apoptosis, with activated Ha-*ras* reducing the incidence of apoptosis over a 48 h period and c-*myc* increasing the incidence of apoptosis over this time (Arends *et al.*, 1993). A similar pattern of high and low levels of apoptosis was seen *in vivo* using solid fibrosarcomas formed by the RBM7 and T1 cell lines (Arends *et al.*, 1994). Increased levels of apoptosis following growth factor withdrawal have also been demonstrated in Rat-1 fibroblasts expressing c-*myc* (Evan *et al.*, 1992) and in murine myeloid cells constitutively expressing c-*myc* (Askew *et al.*, 1991). It was therefore of interest to determine how the expression of these genes affected the mode and rapidity of cell death following irradiation, and whether cellular sensitivity to DNA damage was altered.

### Materials and methods

### *Cell lines: growth conditions and p53 status*

The derivation of the 208F, T1 and RBM7 cell lines used in this study is described elsewhere (Spandidos and Wilkie, 1984; Arends *et al.*, 1993). Cells were grown in the alpha modification of Eagle's medium (ICN/Flow) supplemented with 10% fetal calf serum (Commonwealth Serum Laboratories, Melbourne, Australia). Cells were incubated at 37°C in sealed flasks that had been flushed with 5% carbon dioxide, 5% oxygen and 90% nitrogen. Cells were subcultured by treatment with pronase (Calbiochem). All experiments were performed with asynchronous cell cultures in log-phase growth. Under these conditions, the cell lines had population doubling times of 16 (208F), 16 (T1) and 17 h (RBM7).

The p53 status of each of the cell lines was examined by immunoprecipitation as described in Radford (1994*b*). A

Correspondence: DR Aldridge, Peter MacCallum Cancer Institute, Melbourne 3000, Australia
Received 9 May 1994; revised 2 September 1994; accepted 18 October 1994

positive result was found for extracts of all three cell lines using PAb 421 antibody, which binds to both wild-type and mutant p53 protein, whereas a negative result was found with PAb 240 antibody, which binds specifically to mutant p53 (Gannon et al., 1990).

*γ-Irradiation, $^{125}I$ labelling and clonogenic assay*

Cultures were irradiated at room temperature in a $^{137}Cs$ source at a dose rate of approximately 0.9 Gy $min^{-1}$. Prior to irradiation, cultures were rinsed to remove any dead cells and fresh growth medium was added. Cell monolayers for clonogenic assay were treated with 0.03% pronase in phosphate-buffered saline (PBS) plus 0.2 mM EDTA for 5 min at room temperature and then dispersed by pipetting. After washing, cell suspensions were counted using a Coulter counter and appropriate cell numbers were plated to give 50-100 colonies in each of five replicate Petri dishes. Colonies of $\geqslant 50$ cells were scored after 8 days' incubation at 37°C. Mean ($\pm$ s.e.) cloning efficiencies for 208F, T1 and RBM7 were respectively $0.33 \pm 0.03$, $0.42 \pm 0.05$ and $0.40 \pm 0.04$.

$^{125}I$ labelling was performed by incubating cultures for approximately 24 h in growth medium containing around 2.5 kBq $ml^{-1}$ [$^{125}I$]iododeoxyuridine (NEN/DuPont) and 2.5 μM thymidine. Cells were then washed and incubated in growth medium containing 20 μM thymidine and 20 μM deoxycytidine for 3 h. After chasing, incorporated $^{125}I$ was measured by pelleting cells and counting radioactivity in a Compugamma CS (LKB) gamma counter. Pellets were then resuspended in a known volume and aliquots were taken for counting to determine the number of $^{125}I$ decays per cell per day. Cells were then aliquoted and frozen (at $-1$°C per min in a controlled-rate freezer) in growth medium plus 10% dimethylsulphoxide (DMSO). Cell aliquots were removed from liquid nitrogen storage at various times, after known numbers of $^{125}I$ decays per cell had occurred, and assayed for clonogenic survival. Further details are given in Radford (1991). Freezing and thawing did not significantly affect the cloning efficiency of these cell lines as evidenced by values of $0.33 \pm 0.02$, $0.35 \pm 0.01$ and $0.40 \pm 0.02$ for 208F, T1 and RBM7 respectively.

A least-squares fit, using the criterion $(S_O - S_E)^2/S_O$, where $S_E$ is the estimated survival and $S_O$ is the observed survival for each experimental point, was obtained for cell survival data using the KaleidaGraph (Abelbeck Software) program on a Macintosh computer. The γ-ray survival data were fitted to the equation $S = \exp[-(\alpha D + \beta D^2)]$ where $S$ is survival, $D$ is dose and $\alpha$ and $\beta$ are constants. $^{125}I$-decay survival data were fitted to the simple exponential $S = A_0 \exp(-D/D_0)$, where $S$ is survival, $D$ is the number of $^{125}I$ decays and $A_0$ and $D_0$ are constants.

*Electron microscopy and gel electrophoresis*

At 24 h intervals after irradiation, non-adherent cells were collected by giving flasks several sharp taps, removing the growth medium, and then rinsing the monolayer once with PBS-EDTA. The growth medium and PBS-EDTA wash were pooled and centrifuged and the cell pellets were then fixed on ice for 30 min in growth medium (without serum) containing 0.25% glutaraldehyde and 45 min in 2.5% glutaraldehyde before post fixation with osmium tetroxide. Cells were then embedded in Spurr's resin and sectioned. Sections of control cells were obtained by fixing and embedding cells *in situ* on glass coverslips.

DNA degradation samples were processed as described previously (Radford et al., 1994). Approximately 2 μg of DNA from each sample was electrophoresed on a 1.5% agarose gel using SPP-1/*Eco*RI DNA (Bresatec, Adelaide, Australia) as size markers. Pulsed-field gel electrophoresis (PFGE) was carried out on a CHEF apparatus with a hexagonal array of electrodes (Chu et al., 1986) for 24 h at 150 V with a pulse time of 80 s. Plugs were prepared by mixing $1 \times 10^6$ (unless otherwise stated) non-adherent or adherent cells (removed from flasks by pronase treatment as described above) in growth medium with an equal volume of 1% low gelling temperature agarose (SeaPlaque, FMC) in balanced salt solution (BSS). Plugs were then placed in NDS (10 mM Tris, 0.5 M EDTA, 1% lauroylsarcosine, pH 9.5) plus 1 mg $ml^{-1}$ proteinase K for 60 min on ice followed by overnight incubation at 37°C before electrophoresis. Yeast chromosomes from *Saccharomyces cerevisiae* strain YP148 (Pyle et al., 1988) and λ DNA (BRL) were used as markers. Following electrophoresis, gels were stained with ethidium bromide.

*Measurement of apoptosis, population expansion and mitotic fraction*

Non-adherent cells were collected over 24 h intervals as described above. They were then pelleted and counted using a haemocytometer. The level of apoptosis was determined by mixing equal volumes of non-adherent cell suspension and growth medium containing 10 μg $ml^{-1}$ ethidium bromide and 3 μg $ml^{-1}$ acridine orange and then scoring approximately 500 cells for apoptotic nuclear morphology by fluorescence microscopy. The number of adherent cells present was determined by treating the monolayer with pronase and Coulter counting the suspension. The fraction of non-adherent cells which was apoptotic was multiplied by the total number of non-adherent cells, divided by the total number of cells in both adherent and non-adherent fractions, and then multiplied by 100 to give the percentage apoptosis.

After irradiation, all flasks were rinsed and had fresh growth medium added at 24 h intervals. The non-adherent cells collected thus represented cells released from the monolayer over a 24 h period. A combination of 24 h medium changes and seeding flasks at appropriate cell numbers ensured that cell death was attributable to the effects of irradiation and not to medium depletion. Population expansion was defined as the number of cells in the monolayer at a given time divided by the number at the time of irradiation.

The level of post-irradiation mitotic activity was determined by incubation of cultures with the mitotic spindle poison nocodazole at 0.1 μg $ml^{-1}$ for 3 h and then scoring the fraction of metaphase-arrested cells. Further details are given in Radford and Murphy (1994).

### Results

*Clonogenic survival dose-response is not markedly changed by over-expression of c-*myc *or T24-*ras

Clonogenic survival curves were obtained for the parent and transfected cell lines exposed to either γ-irradiation or DNA-associated $^{125}I$ decays. The γ-ray survival curves suggest that the overexpression of c-*myc* or an activating mutation of Ha-*ras* in these cells does not markedly affect their radiosensitivity (Figure 1). The similarity in the response of these cell lines is particularly evident at high doses. At low doses, the Ha-*ras* transfectant T1 may show increased resistance leading to a slightly larger shoulder region as compared with the parent line. An activated *ras*-induced change in the shoulder region of the survival curve of a transfected line has been reported by others (Hermens and Bentvelzen, 1992).

In order to quantify more readily the level of DNA damage required for cell killing, the sensitivity of the three cell lines to DNA-associated $^{125}I$ decays was measured. Radioactive decay of $^{125}I$ atoms incorporated into cellular DNA produces high linear energy transfer (LET)-type DNA damage (Charlton, 1986) and results in approximately one DNA double-strand break per decay event (Krisch and Sauri, 1975). The $^{125}I$ data shown in Figure 2 suggest that there is no significant difference in the number of DNA double-strand breaks required to produce a lethal event in each of the cell lines. $D_0$ values of $50 \pm 2.5$, $53 \pm 1.4$ and $56 \pm 2.5$ $^{125}I$ decays were obtained for 208F, T1 and RBM7 respectively. These $D_0$ values suggest that around 50-56 $^{125}I$

**Figure 1** Clonogenic survival of 208F (—O—), T1 (--□--) and RBM7 (--◇--) fibroblasts as a function of γ-radiation dose. Data were fitted to a quadratic function of dose as described in Materials and methods. The calculated values of $\alpha(Gy^{-1})$ and $\beta(Gy^{-2})$ were: 208F, 0.40 ± 0.05 and 0.012 ± 0.004; T1, 0.27 ± 0.03 and 0.019 ± 0.003; RBM7, 0.38 ± 0.003 and 0.009 ± 0.004. Each data set was obtained from at least four independent experiments.

**Figure 2** Clonogenic survival curves of 208F (—O—), T1 (--□--) and RBM7 (--◇--) fibroblast cells that had accumulated DNA-associated [125]I decays during liquid nitrogen storage. The data were fitted to a simple exponential function of accumulated decays and $D_0$ values of 50 ± 3, 53 ± 1 and 56 ± 3 [125]I decays were obtained for 208F, T1 and RBM7 respectively. Each data set was obtained from at least three independent experiments.

**Figure 3** Electron micrographs of control (unirradiated) and γ-irradiated (12 Gy plus incubation at 37°C for 24 h) cells. (**a**) 208F control, (**b**) 208F irradiated, (**c**) T1 control, (**d**) T1 irradiated, (**e**) RBM7 control and (**f**) RBM7 irradiated. Scale bars represent 5 μm.

decay-induced DNA double-strand breaks are required to produce a lethal event in each of these cell lines.

*All cell lines used show apoptotic death following irradiation*

The morphology associated with cell death in each of the three cell lines, following exposure to a γ-ray dose (12 Gy) that would reduce clonogenic survival to 0.5% of the control value, was determined. Both light microscopy of stained sections and fluorescence microscopy (after staining with ethidium bromide and acridine orange) suggested that dying cells showed cytoplasmic shrinkage and condensation and margination of nuclear chromatin (data not shown). These features are diagnostic for apoptosis (Arends and Wyllie, 1991). The conclusion that radiation induces apoptosis in each of the three cell lines was confirmed by electron microscopy (Figure 3). These electron micrographs showed characteristic features of apoptosis such as chromatin condensation and margination to the nuclear periphery (Figure 3f), convolutions of the nuclear membrane (Figure 3b) and eventual cellular break-up into apoptotic bodies (Figure 3d). Cells collected over 0–24 or 48–72 h after irradiation were also examined by electron microscopy for features characteristic of necrotic cell death, such as cellular or organelle swelling. Over these time intervals, no evidence indicative of necrotic cell death was found in any of the three cell lines.

The mechanism of cell death after irradiation was further examined by analysis of the pattern of DNA degradation occurring in dying cells. One of the features often associated with apoptosis is the digestion of nuclear DNA into fragments that are multiples of the 180 bp nucleosome unit (Wyllie, 1980; Arends *et al.*, 1990). When electrophoretically separated on an agarose gel, these DNA fragments produce a characteristic 'ladder' pattern. At various time points after irradiation, DNA was extracted from control (non-irradiated) monolayers and separately from the adherent and non-adherent cell fractions of irradiated cultures. The parent line 208F and the c-*myc*-transfected line RBM7 both showed clear DNA ladders characteristic of apoptosis in the non-adherent cell fraction 24 h after irradiation (Figure 4a). However, the Ha-*ras*-transfected line T1 showed no ladder after 24 h (Figure 4a). DNA extracted and analysed from non-adherent T1 cells at 36 or 48 h after irradiation still did not show laddering of the DNA (Figure 4b). DNA was also extracted from all three cell lines at time points prior to 24 h post irradiation, but ladders were not detected (data not shown). DNA degradation in T1 cells was then examined further using PFGE of cells incubated for 24 or 48 h at 37°C after 12 Gy of γ-irradiation. In each case, the DNA from $1 \times 10^6$ cells was examined, except for the 24 h non-adherent fraction where only $0.5 \times 10^6$ cells were available. The adherent fraction of the irradiated cell cultures, and to a lesser extent the control sample, showed a broad range of DNA sizes, indicating random degradation. DNA from the non-adherent fraction of irradiated T1 cultures incubated for 48 h, showed more marked degradation and the possible appearance of a 100 kbp intermediate that is subsequently degraded further (Figure 4c).

*Elevated expression of* c-myc *increases the incidence of apoptosis following γ-irradiation*

Previous work using these cell lines had shown a difference in the incidence of apoptosis at 48 h after serum withdrawal (Arends *et al.*, 1993). It was therefore of interest to examine the relative time of onset of apoptosis for asynchronous cultures of each of the cell lines following a 0.5% clonogenic survival γ-ray dose. At 24 h periods following irradiation, non-adherent cells were collected, counted, and the percentage of cells undergoing apoptotic death was scored by fluorescence microscopy. Apoptotic cells were not found in adherent cell populations and non-adherent, non-apoptotic cells were found to account for less than 3.5% of non-apoptotic cells. The results of this experiment showed that the c-*myc*-transfected cell line RBM7 had a markedly greater incidence

**Figure 4** Ethidium bromide-stained agarose gel separations, using conventional (**a** and **b**) or pulsed-field gel electrophoresis (**c**) of DNA extracted from the control (C), irradiated adherent cells (Ad) and irradiated non-adherent cells (N). Cells were incubated at 37°C after γ-irradiation (12 Gy) for the indicated time periods.

of radiation-induced apoptosis, over the time interval examined, than the other lines (Figure 5a). However, the expression of activated Ha-*ras* (T1) did not alter the frequency of apoptosis compared with the parent line (208F) up to 96 h post irradiation (Figure 5a). Adherent cells were also counted and RBM7 was found to maintain a similar number of cells on the monolayer as the parent line in spite of its higher rate of apoptosis. Cell numbers of T1, however, continued to increase up to 72 h post irradiation (Figure 5b).

**Figure 5** (**a**) Fraction of cell population showing apoptotic morphology (determined by fluorescence microscopy of ethidium bromide/acridine orange-stained cells) after γ-irradiation (12 Gy) and incubation at 37°C for the indicated time periods. ☐, 208F; ■, T1; ▨, RBM7 (**b**). Increase in adherent cell numbers after γ-irradiation (12 Gy) relative to number present at the time of irradiation. (**c**) Entry of γ-irradiated (12 Gy) cell populations into mitosis as determined by incubation with nocodazole for successive 3 h intervals. Data points represent the mean (± s.d.) of two determinations. Error bars have been omitted from **c** for the sake of clarity. —O—, 208F; --■--, T1; --◇--, RBM7.

In order to determine the level and time of resumption of mitotic activity, replicate cultures of the three cell lines were incubated with the mitotic spindle poison nocodazole for successive 3 h intervals following 12 Gy of γ-irradiation (Figure 5c). Following an initial marked depression of mitotic activity, a wave of mitosis was noted, which suggested a partial cell cycle-synchronising effect of irradiation on each of the lines. Cultures of both the *myc*- and *ras*-transfected lines (RBM7 and T1) resumed cycling more rapidly and showed a higher fraction of cells entering mitosis than the parental 208F line. *In toto*, the data in Figure 5 suggest that, relative to the parental cell line, the level of apoptosis per mitosis is similar in the *myc*-transfected line but might be decreased in the *ras*-transfected line. Assuming that radiation-induced apoptosis occurs after mitosis in these cell lines, the increased incidence of apoptosis in the *myc*-transfected line would then be a consequence of its higher (relative to the parental line) level of post-irradiation mitotic activity. Time-lapse cinemicroscopy studies will be required in order to confirm these conclusions.

### Discussion

The 208F rat fibroblast line and its *myc*- and *ras*-transfected derivatives RBM7 and T1 all undergo radiation-induced apoptosis as evidenced by morphology. This response is broadly in keeping with previous observations of 'spontaneous' cell death of these cell lines in culture by apoptosis, (albeit at high and low levels respectively for RBM7 and T1) (Arends *et al.*, 1993) and also the pattern of death observed in solid tumours: RBM7 fibrosarcomas showed a high level of apoptosis and very little necrosis, but T1 demonstrated widespread necrosis with low levels of apoptosis (Arends *et al.*, 1994). This occurrence of radiation-induced apoptosis in all three fibroblast lines examined in this study differs from some reports in which normal and transformed fibroblasts were found to undergo necrosis after irradiation (e.g. Afanas'ev *et al.*, 1986; Radford, 1991). However, Tomei *et al.* (1988) using C3H-10T1/2 mouse fibroblasts and Lowe *et al.* (1988) using adenovirus E1A gene-expressing mouse embryonic fibroblasts both reported induction of apoptosis after irradiation.

In an effort to explain differences in response to irradiation between cell types, many groups have looked at the possible link between oncogene expression and radiosensitivity (reviewed in Kasid *et al.*, 1993). The consequences of transfection of members of the *ras* family of oncogenes have been widely studied. However, these studies have yielded conflicting results. Expression of mutationally activated *ras* or overexpression of normal *ras* have been reported to increase the radioresistance of mouse 3T3 fibroblasts and of rat rhabdomyosarcoma cells (Sklar, 1988; Samid *et al.*, 1991; Hermens and Bentvelzen, 1992), while studies using transfected normal or immortalised human cells found that expression of activated *ras* does not, by itself, lead to an increase in radioresistance (Mendonca *et al.*, 1991; Su and Little, 1992). We found that expression of activated Ha-*ras* in the T1 derivative of the 208F fibroblast line had no marked effect on its clonogenic survival dose–responses for γ-ray or $^{125}I$ decay-induced DNA damage. Activated *ras* did not appear to alter the fraction of the total cell population undergoing apoptosis up to 96 h after irradiation as compared with the parent line. However, irradiated cultures of the T1 line showed greater residual proliferation, suggesting that activated *ras* protein may decrease the level of apoptosis per mitosis. This effect may be related to the inhibition of serum withdrawal-induced apoptosis and to the decrease in susceptibility to apoptosis under both *in vitro* and *in vivo* growth conditions observed previously for this cell line (Arends *et al.*, 1993, 1994).

The expression of activated Ha-*ras* inhibited the endonuclease activity responsible for the 180 bp nucleosomal ladder that is commonly associated with apoptosis (Arends *et al.*, 1990). This finding is consistent with that of Arends *et al.*

(1993), who showed that this endonuclease(s) is down-regulated by the expression of activated *ras*. Despite lacking detectable nucleosomal ladder-producing endonuclease activity, irradiated T1 cells showed extensive DNA degradation to relatively high molecular weight fragments and normal apoptotic morphology. Apoptosis without the appearance of nucleosomal ladders has been reported previously (Cohen *et al.*, 1992), and it has been shown that the characteristic apoptotic nuclear morphology is associated with initial cleavage of nuclear DNA into 300 kbp fragments and then into 50 kbp fragments and does not require the production of 180 bp nucleosomal ladders (Brown *et al.*, 1993; Oberhammer *et al.*, 1993).

The RBM7 cell line, which overexpresses c-*myc*, showed an increase in the incidence of radiation-induced apoptosis, over the time period examined, with respect to the parent line. This finding is consistent with the observations of Arends *et al.* (1993, 1994) that the RBM7 line shows increased susceptibility to serum withdrawal-induced apoptosis and increased intrinsic apoptosis when growing as a solid fibrosarcoma or in culture, and with the hypothesis that c-*myc* expression induces a state in which cells are 'primed' for apoptosis (Arends and Wyllie, 1991). The increase in apoptosis may reflect a more rapid post-irradiation resumption of mitotic activity in the RBM7 cell line.

However, despite their increased rate of $\gamma$-irradiation-induced apoptotic death, the sensitivity of RBM7 cells to either $\gamma$-irradiation or DNA-associated $^{125}I$ decay-induced cell killing is not significantly different from that of the parent line. This result contrasts with previous data from mouse lymphoid lines, which revealed a correlation between rate of radiation-induced apoptotic death and radiosensitivity (Radford, 1994*a*). However, it should be noted that, even in the RBM7 line, apoptosis is induced considerably more slowly in irradiated fibroblasts than in the more radiosensitive lymphoid lines. For example, apoptotic cells were not detected in RBM7 cultures until at least 8 h after irradiation (data not shown), as compared with 1–2 h in radiosensitive lymphoid lines (Radford, 1994*a*). The comparatively lengthy time period available to irradiated RBM7 cells, prior to possible induction of apoptosis, may be adequate for DNA repair. The reason for the difference in the rate of induction of apoptosis between irradiated fibroblasts and some lymphoid lines is currently unclear. Studies with mouse lymphoid lines have suggested that rapid induction of apoptosis after irradiation is dependent upon the presence of wild-type p53 protein (Radford, 1994*b*). Although all three fibroblast lines used in this study appear to contain non-mutant p53 protein, a definitive conclusion awaits DNA sequencing (see Materials and methods). Studies by other investigators, although not defining the mode of cell death occurring, have also generally concluded that overexpression of c-*myc* does not alter radioresistance (reviewed in Kasid *et al.*, 1993).

These findings lead us to question the hypothesis that a cell's radiosensitivity can be directly related to its mode of death. Indeed, the number of $^{125}I$ decay-induced DNA double-strand breaks required to produce a lethal event in the 208F line and its transfected derivatives was similar to the number required to kill the V79 fibroblast line ($D_0 = 61 \pm 2$), which undergoes necrotic cell death (Radford, 1991). This suggests that differences in radiosensitivity between cell lines may be related more to the intrinsic characteristics of the cell type of origin than to mode of death and that radiosensitivity is a phenotypic property distinct from susceptibility to apoptosis and that it may be independently genetically influenced. However, there is a need to study the relationship between radiosensitivity and mode of cell death in a wider range of cell types before such conclusions can be confirmed. It should also be noted that the significant difference in residual post-irradiation proliferation between the cell lines studied may complicate the extrapolation of our results to the *in vivo* situation.

**Acknowledgements**
DA was supported by a Peter MacCallum Cancer Institute PhD studentship and the work was funded in part by a grant from the Anti-Cancer Council of Victoria. The authors are grateful to Dr John Radley and Manuela Palatsides for the production of electron micrographs and to Dr Alan Harris (Walter and Eliza Hall Institute of Medical Research) for reading the manuscript.

### References

AFANAS'EV VN, KOROL BA, MANTSYGIN YA, NELIPOVICH PA, PECHATNIKOV VA AND UMANSKY SR. (1986). Flow cytometry and DNA degradation characteristic of two types of cell death. *FEBS Lett.*, **194**, 347–350.

ARENDS MJ AND WYLLIE AH. (1991). Apoptosis: mechanisms and roles in pathology. *Int. Rev. Exp. Pathol.*, **32**, 223–251.

ARENDS MJ, MORRIS RG AND WYLLIE AH. (1990). Apoptosis: the role of the endonuclease. *Am. J. Pathol.*, **136**, 593–608.

ARENDS MJ, McGREGOR AH, TOFT NJ, BROWN EJH AND WYLLIE AH. (1993). Susceptibility to apoptosis is differentially regulated by c-*myc* and mutated Ha-*ras* oncogenes and is associated with endonuclease availability. *Br. J. Cancer*, **68**, 1127–1133.

ARENDS MJ, McGREGOR AH AND WYLLIE AH. (1994). Apoptosis is inversely related to necrosis and determines net growth in tumours bearing constitutively expressed myc, ras and HPV oncogenes. *Am. J. Pathol.*, **144**, 1045–1057.

ASKEW DS, ASHMUN RA, SIMMONS BC AND CLEVELAND JL. (1991). Constitutive c-*myc* expression in an IL-3-dependent myeloid cell line suppresses cell cycle arrest and accelerates apoptosis. *Oncogene*, **6**, 1915–1922.

BURNET NG, NYMAN J, TURESSON I, WURM R, YARNOLD JR AND PEACOCK JH. (1992). Prediction of normal-tissue tolerance to radiotherapy from *in-vitro* cellular radiation sensitivity. *Lancet*, **339**, 1570–1571.

BROWN DG, SUN XM AND COHEN GM. (1993). Dexamethasone-induced apoptosis involves cleavage of DNA to large fragments prior to internucleosomal fragmentation. *J. Biol. Chem.*, **268**, 3037–3039.

CHARLTON DE. (1986). The range of high LET effects from $^{125}I$ decays. *Radiat. Res.*, **107**, 163–171.

CHU G, VOLLRA TH D AND DAVIS RW. (1986). Separation of large DNA molecules by contour-clamped homogeneous electrical fields. *Science*, **234**, 1582–1585.

COHEN GM, SUN XM, SNOWDEN RT, DINSDALE D AND SKILLETER DN. (1992). Key morphological features of apoptosis may occur in the absence of internucleosomal DNA fragmentation. *Biochem. J.*, **286**, 331–334.

EVAN GI, WYLLIE AH, GILBERT CS, LITTLEWOOD TD, LAND H, BROOKS M, WALTERS CM, PENN LZ AND HANCOCK DC. (1992). Induction of apoptosis in fibroblasts by c-myc protein. *Cell*, **69**, 119–128.

GANNON JV, GREAVES R, IGGO R AND LANE DP. (1990). Activating mutations in p53 produce a common conformational effect. A monoclonal antibody specific for the mutant form. *EMBO J.*, **9**, 1595–1602.

HENDRY JH. (1985). Review: survival curves for normal-tissue clonogens: a comparison of assessments using *in vitro*, transplantation, or *in situ* techniques. *Int. J. Radiat. Biol.*, **47**, 3–16.

HERMENS AF AND BENTVELZEN PAJ. (1992). Influence of the H-*ras* oncogene on radiation response of a rat rhabdomyosarcoma cell line. *Cancer Res.*, **52**, 3073–3082.

KASID U, PIROLLO K, DRITSCHILO A AND CHANG E. (1993). Oncogenic basis of radiation resistance. *Adv. Cancer Res.*, **61**, 195–233.

KRISCH RE AND SAURI CJ. (1975). Further studies of DNA damage and lethality from the decay of iodine-125 in bacteriophages. *Int. J. Radiat. Biol.*, **27**, 553–560.

LOWE SW, RULEY HE, JACKS T AND HOUSMAN DE. (1993). p53-dependent apoptosis modulates the cytotoxicity of anticancer agents. *Cell*, **74**, 957–967.

MENDONCA MS, BOUKAMP P, STANBRIDGE EJ AND REDPATH JL. (1991). The radiosensitivity of human keratinocytes: influence of activated c-H-ras oncogene expression and tumorigenicity. *Int. J. Radiat. Biol.*, **59**, 1195–1206.

MEYN RE, STEPHENS LC, ANG KK, HUNTER NR, BROCK WA, MILAS L AND PETERS LJ. (1993). Heterogeneity in the development of apoptosis in irradiated murine tumours of different histologies. *Int. J. Radiat. Biol.*, **64**, 583–591.

OBERHAMMER F, WILSON JW, DIVE C, MORRIS ID, HICKMAN JA, WAKELING AE, WALKER PR AND SIKORSKA M. (1993). Apoptotic death in epithelial cells: cleavage of DNA to 300 and/or 50 kb fragments prior to or in the absence of internucleosomal fragmentation. *EMBO J.*, **12**, 3679–3684.

PYLE LE, CORCORAN LN, COCKS BG, BERGEMANN AD, WHITLEY JC AND FINCH LR. (1988). Pulsed-field electrophoresis indicates larger-than-expected sizes for mycoplasma genomes. *Nucleic Acids Res.*, **16**, 6015–6025.

RADFORD IR. (1991). Mouse lymphoma cells that undergo interphase death show markedly increased sensitivity to radiation-induced DNA double-strand breakage as compared with cells that undergo mitotic death. *Int. J. Radiat. Biol.*, **59**, 1353–1369.

RADFORD IR. (1994*a*). Radiation response of mouse lymphoid and myeloid cell lines. Part I. Sensitivity to killing by ionizing radiation, rate of loss of viability, and cell type of origin. *Int. J. Radiat. Biol.*, **65**, 203–215.

RADFORD IR. (1994*b*). p53 status, DNA double-strand break repair proficiency and radiation response of mouse lymphoid and myeloid cell lines. *Int. J. Radiat. Biol.* (in press).

RADFORD IR AND MURPHY TK. (1994). Radiation response of mouse lymphoid and myeloid cell lines. III. Different signals can lead to apoptosis and may influence sensitivity to killing by DNA double strand breakage. *Int. J. Radiat. Biol.*, **65**, 229–239.

RADFORD IR, MURPHY TK, RADLEY JM AND ELLIS SL. (1994). Radiation response of mouse lymphoid and myeloid cell lines. II. Apoptotic death is shown by all lines examined. *Int. J. Radiat. Biol.*, **65**, 217–227.

ROFSTAD EK. (1986). Radiation biology of malignant melanoma. *Acta Radiol. Oncol.*, **25**, 1–10.

SAMID D, MILLER AC, RIMOLDI D, GAFNER J AND CLARK EP. (1991). Increased radiation resistance in transformed and non-transformed cells with elevated ras proto-oncogene expression. *Radiat. Res.*, **126**, 244–250.

SKLAR MD. (1988). The *ras* oncogenes increase the intrinsic resistance of NIH 3T3 cells to ionizing radiation. *Science*, **239**, 645–647.

SPANDIDOS DA AND WILKIE NM. (1984). Malignant transformation of early passage rodent cells by a single mutant human oncogene. *Nature*, **310**, 469–475.

STEEL GG. (1989). Radiobiology of human tumour cells. In *The Biological Basis of Radiotherapy*, 2nd edn. Steel GG, Adams GE and Horwich A. (eds) pp. 163–179. Elsevier: Amsterdam.

SU LN AND LITTLE JB. (1992). Transformation and radiosensitivity of human diploid skin fibroblasts transfected with activated ras oncogene and SV40 T-antigen. *Int. J. Radiat. Biol.*, **62**, 201–210.

TOMEI LD, KANTER P AND WENNER CE. (1988). Inhibition of radiation-induced apoptosis *in vitro* by tumor promoters. *Biochem. Biophys. Res. Commun.*, **155**, 324–331.

WYLLIE AH. (1980). Glucocorticoid-induced thymocyte apoptosis is associated with endogenous endonuclease activation. *Nature*, **284**. 555–556.